# Factors Affecting Intensive Aflibercept Treatment Response in Diabetic Macular Edema: A Real-World Study

**DOI:** 10.1155/2023/1485059

**Published:** 2023-07-18

**Authors:** Ye Eun Han, Jaehyuck Jo, Yoon Jeon Kim, Junyeop Lee

**Affiliations:** ^1^Department of Ophthalmology, Asan Medical Center, University of Ulsan, College of Medicine, Seoul, Republic of Korea; ^2^Asan Diabetes Center, Asan Medical Center, Seoul, Republic of Korea

## Abstract

**Objective:**

To investigate the systemic and ocular factors that affect the response to intensive aflibercept treatment in diabetic macular edema (DME) in a real-world setting.

**Methods:**

This retrospective cohort study evaluated 30 eyes of 23 patients with DME who underwent intensive intravitreal aflibercept injections (five monthly loading doses). Treatment response was assessed by central retinal thickness (CRT) and best-corrected visual acuity (BCVA) at each monthly visit. The patients were categorized as good (<300 *μ*m) and suboptimal (≥300 *μ*m) responders based on CRT after the loading phase. Baseline systemic and ocular factors associated with treatment response were investigated.

**Results:**

The mean CRT and BCVA significantly improved after five loading injections (486.87 ± 95.46 to 334.90 ± 69.47 *μ*m and 0.51 ± 0.30 to 0.35 ± 0.25 LogMAR, respectively, all *p* < 0.05). During 12 months of follow-up, 16 eyes (53.33%) maintained CRT without additional treatment. Eyes with diabetes mellitus (DM) for ≥15 years, estimated glomerular filtration rate (eGFR) < 80 mL/min/1.73 m^2^, serum creatinine ≥ 0.95 mg/dL and potassium ≥ 4.7 mmol/L, and presence of epiretinal membrane (ERM) were more likely to have a suboptimal response to the treatment.

**Conclusions:**

Five monthly loading doses of intravitreal aflibercept injection provided significant anatomical and visual improvements in patients with DME. Patients with longer DM duration, lower eGFR, higher serum creatinine or potassium levels, or ERM were predisposed to a suboptimal treatment response. Individual response to intensive aflibercept treatment for DME can be predicted by these systemic and ocular risk factors.

## 1. Introduction

Diabetic macular edema (DME) is the most common cause of visual deterioration in patients with diabetic retinopathy [1]. The main features of DME are disruption of the blood-retinal barrier and increased vascular permeability, causing abnormal fluid accumulation in the intraretinal layers of the macula [2]. Vascular endothelial growth factor (VEGF) is the key mediator of the pathophysiology of DME, and thus, intravitreal anti-VEGF injections have become the mainstay of treatment for center-involved DME [3]. Aflibercept is the only antiangiogenic drug that blocks all isoforms of VEGF and placental growth factor (one of the VEGF families) with the highest affinity and the longest half-time [2]. Although no consensus has been established on the aflibercept treatment regimen, intensive initial loading injections followed by as-needed (pro re nata (PRN)) doses are considered beneficial to improve treatment efficacy and alleviate treatment burden [4–6].

DRCR.net Protocol T study [5], a randomized controlled pivotal trial, demonstrated that monthly aflibercept injection as a loading regimen was more effective in resolving DME than bevacizumab or ranibizumab. However, persistent DME was present in 31.6% of eyes after the initial six monthly aflibercept injections [7]. In fact, predicting the individual response to intensive aflibercept treatment is challenging in real-world practice, especially because Protocol T excluded the eyes with systemic diseases except for diabetes (i.e., significant renal, hypertensive, or cardiovascular disease) or previous DME treatment. To maximize the therapeutic efficacy of intensive aflibercept, modifiable risk factors that affect the treatment response need to be clarified.

The influence of systemic factors on diabetic retinopathy has been well studied. Previous studies have shown that control of hyperglycemia, hypertension, serum cholesterol, and renal function can significantly delay the onset and progression of diabetic retinopathy [8–12]. However, there is no study regarding systemic and ocular factors influencing the response to intensive aflibercept monotherapy in DME, although there are some studies for other anti-VEGFs (bevacizumab and ranibizumab) [13–24]. Therefore, this study aimed to evaluate the treatment outcomes of intensive aflibercept treatment in DME and investigate the modifiable systemic and ocular factors that affect the treatment response in a real-world setting.

## 2. Materials and Methods

### 2.1. Patients

A retrospective cohort study was conducted at Asan Medical Center, a tertiary referral center in Seoul, South Korea, from April 2020 to January 2022. We retrospectively reviewed the medical records of all consecutive DME patients who received their first intensive intravitreal aflibercept injections (five monthly loading doses)+pro re nata (PRN) treatment. Korean Health Insurance started its coverage for aflibercept (the first five consecutive injections, followed by bimonthly injections up to 14 times) for DME treatment since April 2020. According to the Korean Health Insurance guideline, only those with a baseline central retinal thickness (CRT) ≥ 300 *μ*m with HbA1c < 8.0% were advised to receive the treatment. Eyes were excluded if the systemic and ophthalmologic evaluations had not been fully performed or if they had a history of other ocular diseases that possibly caused macular edema (i.e., retinal vein occlusion, age-related macular degeneration, or intraocular inflammation). This study was approved by the Institutional Review Board of the Asan Medical Center and the University of Ulsan College of Medicine, Seoul, South Korea, and adhered to the tenets outlined in the Declaration of Helsinki (IRB number: 2021-1560). Informed consent for intravitreal injection was obtained from all patients; however, informed consent for the study was waived by the IRB of Asan Medical Center due to the study's retrospective nature.

### 2.2. Systemic Evaluation

We reviewed the past medical history and baseline blood test results, including glycated hemoglobin (HbA1c), complete blood count (CBC: hemoglobin, white blood cells, and platelets), renal function tests (serum creatinine and estimated glomerular filtration rate (eGFR, in mL/min/1.73 m^2^) according to the Chronic Kidney Disease Epidemiology Collaboration equation [25]), liver function test (LFT: aspartate aminotransferase test [25] and alanine aminotransferase test), serum electrolytes (calcium, sodium, potassium, and chloride), and albumin, conducted before the treatment initiation.

### 2.3. Ocular Evaluation

All patients underwent a complete ophthalmologic evaluation, including a comprehensive review of ophthalmologic history, best-corrected visual acuity (BCVA, measured by the Snellen chart, then converted to LogMAR), slit-lamp biomicroscopy, ultra-wide-field fluorescence angiography (Heidelberg Retinal Angiograph-2; Heidelberg Engineering, Heidelberg, Germany), spectral domain optical coherence tomography (SD-OCT; Heidelberg Engineering, Heidelberg, Germany), and funduscopic examinations through dilated pupils by retinal specialists (Y.J.K and J.L.). The diagnosis of DME and (non)proliferative diabetic retinopathy ((N)PDR) was based on the criteria of the Early Treatment Diabetic Retinopathy Study (ETDRS) [26]. DME was quantified by CRT, the average retinal thickness (distance between the internal limiting membrane and the retinal pigment epithelium) of ETDRS central subfield (fovea center with 1 mm diameter). CRT was measured by SD-OCT horizontal raster pattern scan of the macula centered on the fovea (20° × 20°, 5.4 mm × 5.4 mm field) and automatically calculated by built-in software (version 1.10.2.0). The evaluation of the OCT images was performed by two independent examiners (Y.E.H. and J.J.). For the analysis, OCT images with poor quality or artifacts (e.g., segmentation error, motion artifacts, or decentration) were excluded.

### 2.4. Treatment Procedures

The patients were scheduled to receive five consecutive monthly intravitreal aflibercept injections (Eylea; Bayer Inc., 2 mg/0.05 mL each at baseline, months 1, 2, 3, and 4) as the loading phase. All patients were followed up monthly after the loading phase and received as-needed (PRN) treatment only if the CRT was ≥300 *μ*m and increased to >50 *μ*m compared with the previous measurement [27]. The PRN treatment was not limited to aflibercept monotherapy but could be switched to alternative treatments (i.e., bevacizumab (Avastin, Genentech Inc.), dexamethasone intravitreal implant (Ozurdex, Allergan plc, Dublin, Ireland), or subtenon triamcinolone injection) as the most suitable treatment for a given ocular condition.

### 2.5. Evaluation of Treatment Outcomes and Factors Affecting Treatment Response

The anatomical and functional treatment response was monitored by CRT and BCVA in every monthly visit. The primary treatment outcome of the intensive aflibercept treatment was determined by (1) mean CRT and BCVA changes and (2) the proportion of good responders (<300 *μ*m) and suboptimal responders (≥300 *μ*m) based on CRT after completion of five monthly loading injections (at month 5) [7, 28]. We also evaluated (3) the proportion of the eyes that maintained their CRT without requiring additional treatment during the PRN regimen to assess the durability of the aflibercept loading injections. To investigate factors affecting treatment response, we compared the baseline ocular and systemic factors between good and suboptimal responders. We assessed changes in CRT and BCVA according to each significant factor.

### 2.6. Statistical Analysis

Repeated measure analysis of variance (ANOVA) was used to determine whether CRT and BCVA are significantly changed compared with their baseline values. The Kaplan-Meier analysis was used to assess the maintenance of the therapeutic effect of aflibercept loading injections during the maintenance phase. A Mann–Whitney *U*-test and Wilcoxon signed-rank test were used to compare the systemic and ocular factors between groups depending on the variable types. Logistic regression analysis was used to estimate the association between suboptimal treatment response and systemic and ocular factors. The receiver operation characteristic (ROC) curve was used to determine the cutoff value of the risk factors for suboptimal treatment response. The point on the curve with the maximum sensitivity and specificity was selected as the cutoff value [29]. A *p* value of less than 0.05 was considered statistically significant. All statistical analyses were performed using SPSS software version 21.0 (IBM Corp).

## 3. Results

### 3.1. Baseline Characteristics

A total of 30 eyes from 23 patients with DME were evaluated. Their baseline characteristics are shown in Table 1. The mean age was 60.70 ± 9.28 with a male-to-female ratio of 19 : 11. The mean duration of diabetes mellitus (DM) was 14.33 ± 8.73 years, and HbA1c was 6.93 ± 1.52% (under 13.33% of insulin treatment and 86.66% of oral hypoglycemic agent medication alone). Eighteen eyes (60%) were diagnosed with NPDR and 12 eyes (40%) with PDR. Eight eyes (26.66%) were treatment-naïve, while others had previous treatment histories (4 eyes (13.33%) with vitrectomy and panretinal photocoagulation (PRP), 5 eyes (16.66%) with bevacizumab injection and PRP, 5 eyes (16.66%) with bevacizumab injection only, and 8 eyes (26.66%) with PRP only).

### 3.2. Treatment Outcomes

After completion of loading injection (at month 5), the mean CRT and BCVA were significantly improved (486.87 ± 95.46 to 334.90 ± 69.47 *μ*m and 0.51 ± 0.30 to 0.35 ± 0.25 LogMAR, all *p* < 0.05). According to CRT at month 5, eleven eyes (36.66%) were good responders, and 19 eyes (63.33%) were suboptimal responders.

**Figure 1:**
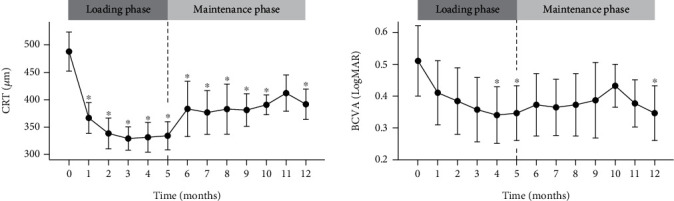
Mean changes in central retinal thickness and best-corrected visual acuity in the whole cohort. Mean changes in central retinal thickness (CRT) and best-corrected visual acuity (BCVA) with 95% confidence interval error bars during the loading phase (months 0-4) and maintenance phase (PRN regimen, months 5-12). For month five, the result of the five monthly loading aflibercept injections is indicated by the vertical black dotted line. (^∗^significant difference compared to the baseline value in repeated measures ANOVA, *p* < 0.05).

**Figure 2:**
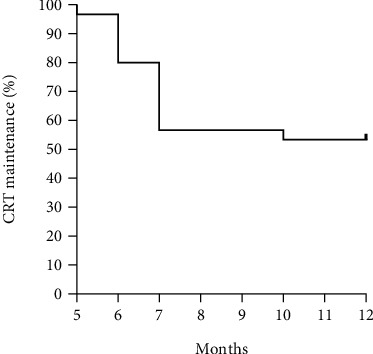
Kaplan-Meier curve for central retinal thickness maintenance after the loading phase. Percent probability of maintaining central retinal thickness (CRT) without additional treatment during maintenance phase (PRN regimen, months 5-12).

During the PRN regimen (maintenance phase), the mean CRT increased along with the deterioration of BCVA, although it was not worse than the baseline values (Table 2 and Figure 1). Sixteen eyes (53.33%) could maintain the improved CRT without additional treatment; however, 14 eyes (46.66%) needed PRN treatment during 12 months of follow-up (at month 5 (1 eye, 3.33%), month 6 (5 eyes, 16.67%), month 7 (7 eyes, 23.33%), and month 10 (1 eye, 3.33%), respectively) (Figure 2). Among those, 7 eyes (23.33%) received aflibercept monotherapy, while 7 eyes (23.33%) switched to other therapies (2 eyes (6.66%) to Ozurdex and bevacizumab, 1 eye (3.33%) to Ozurdex and subtenon triamcinolone injection, 3 eyes (10.00%) to Ozurdex only, and 1 eye (3.33%) to bevacizumab only). During the entire cohort period, intensive aflibercept treatment did not cause any serious ocular or nonocular complications, such as intraocular inflammation and cardiovascular events that would require treatment discontinuation.

### 3.3. Risk Factors for Suboptimal Treatment Response

**Figure 3:**
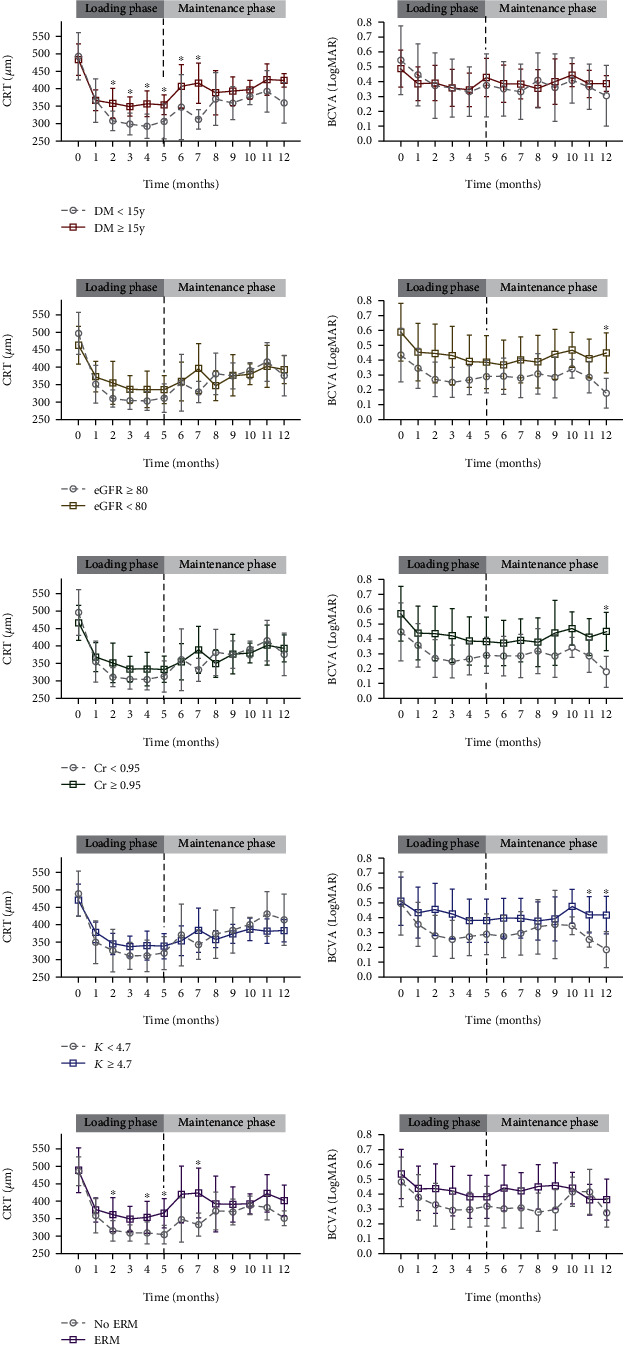
Mean changes in central retinal thickness and best-corrected visual acuity according to systemic and ocular risk factors for suboptimal treatment response. Mean changes in central retinal thickness (CRT, left) and best-corrected visual acuity (BCVA, right) during the loading phase (months 0-4) and maintenance phase (PRN regimen, months 5-12) with 95% confidence interval error bars in (a) diabetic mellitus (DM) duration ≥ 15 years (red solid line) vs. <15 years (gray dotted line); (b) estimated glomerular filtration rate (eGFR) < 80 mL/min/1.73 m^2^ (brown solid line) vs. ≥80 mL/min/1.73 m^2^ (gray dotted line); (c) serum creatinine (Cr) ≥ 0.95 mg/dL (green solid line) vs. <0.95 mg/dL (gray dotted line); (d) potassium (K) ≥ 4.7 mmol/L (blue solid line) vs. <4.7 mmol/L (gray dotted line); and (e) presence of epiretinal membrane (ERM, purple solid line) vs. absence of ERM (gray dotted line). For month five, the result of the five monthly loading aflibercept injections is indicated by the vertical black dotted line (^∗^significant difference between groups in Mann–Whitney *U*-test, *p* < 0.05).

Suboptimal responders had significantly longer DM duration (17.42 ± 8.25 vs. 9.00 ± 7.04 years, *p* = 0.008), lower eGFR (53.80 ± 34.16 vs. 85.00 ± 25.88 mL/min/1.73 m^2^, *p* = 0.018), higher serum creatinine (2.23 ± 1.98 vs. 1.03 ± 0.92 mg/dL, *p* = 0.049) and potassium (4.89 ± 0.60 vs. 4.20 ± 0.51 mmol/L, *p* = 0.004) levels, and higher prevalence of epiretinal membrane (ERM) (68.42% vs. 18.18%, *p* = 0.008) compared with good responders. Other systemic or ocular factors (age, sex, HbA1c, DM medication, CBC, LFT, serum electrolytes, albumin, hypertension, hyperlipidemia, baseline CRT and BCVA, severity of DM retinopathy, and previous ocular treatment history) did not show significant differences between the two groups (Table 3). According to the ROC curve, the cutoff value of each significant risk factor for suboptimal treatment response was as follows: DM duration ≥ 15 years, eGFR < 80 mL/min/1.73 m^2^, serum creatinine ≥ 0.95 mg/dL, and potassium ≥ 4.7 mmol/L. Logistic regression analysis revealed that eyes with DM duration ≥ 15 years (odds ratio (OR), 9.33; *p* = 0.011), eGFR < 80 mL/min/1.73 m^2^ (OR, 7.35; *p* = 0.046), serum creatinine ≥ 0.95 mg/dL (OR, 7.33; *p* = 0.026), potassium ≥ 4.7 mmol/L (OR, 5.87; *p* = 0.041), and ERM (OR, 9.75; *p* = 0.014) were more likely to have a suboptimal treatment response (Supplementary Table 1). When we tracked the longitudinal changes in CRT and BCVA during 12 months of follow-up according to these cutoff values (Figure 3), eyes with longer DM duration, lower eGFR, higher serum creatinine and potassium levels, and ERM showed less decreased CRT in response to aflibercept treatment. The trend of BCVA changes was similar to that of CRT throughout the follow-up period, although they are not completely consistent, and BCVA changes showed greater fluctuations (Figure 3).

## 4. Discussion

This study demonstrated that intensive aflibercept treatment provides a significant anatomical and functional improvements in DME patients. More importantly, eyes with longer DM duration, lower eGFR, higher serum creatinine and potassium levels, and ERM were associated with a suboptimal treatment response. To the best of our knowledge, this is the first study to investigate systemic and ocular factors that affect the intensive aflibercept treatment outcomes in DME patients in a real-world setting.

Previous clinical trials have found the importance of intensive initial aflibercept loading injections for DME treatment. In Protocol T [5], most patients required more than six initial monthly injections. The VIVID and VISTA studies [30] demonstrated that the five monthly loading doses are significantly superior in functional and anatomical improvements over PRP treatment. Post hoc analysis [6] of VIVID and VISTA reported that anatomical and functional improvements continued after the fourth and fifth loading injections. This study also found that five aflibercept loading injections provide significant anatomical and visual improvements. Furthermore, we found that 53.33% of eyes maintained CRT after the loading phase without additional treatment during 12 months of follow-up.

However, after five loading injections, DME persisted in more than half of the eyes (63.33%) in this study. A post hoc analysis of Protocol T [7] demonstrated that persistent DME was present in 31.6% of the eyes after the initial six monthly aflibercept injections, although its presence is less likely after bevacizumab. Although the criteria of “persistent” were not the same, these results suggest that the proportion of persistent DME in the real world seems to be higher than that in clinical trials. Therefore, more than five monthly doses of intensive initial treatment may be required in a specific subgroup of patients to achieve satisfactory resoluation of DME. Although the first injection showed the greatest improvement in both CRT and BCVA, continuous improvement was observed as the number of injections increased during the loading phase. Three eyes (10%) with a suboptimal response after the third injection changed into good responders after the fifth injection (delayed responders, data not shown). It is also worth noting that, after discontinuation of monthly loading injections, CRT increased and did not fully recover to the loading phase level despite PRN treatment. VIVID and VISTA studies [30] showed that after the loading phase, monthly fixed aflibercept injection groups demonstrated continuous CRT reduction throughout 1 year of the following period. Consequently, their 1-year mean reduction in CRT was greater than that observed in our study. Additionally, a treat-and-extend regimen of aflibercept in DME demonstrated a 2-year efficacy comparable to that of a fixed-dose regimen [31]. Meanwhile, in Protocol U [32], an alternative dexamethasone implant was not beneficial in combination with continuous ranibizumab injections for persistent DME with at least three monthly anti-VEGF injections. Therefore, to maintain DME improvement after the loading phase, a proactive aflibercept injection regimen, such as a fixed-dose or treat-and-extend strategy, is required, rather than PRN or dexamethasone combination treatment.

Several studies have reported the association between systemic factors and DME treatment response; however, their results were inconsistent and mainly dealt with ranibizumab or bevacizumab [15, 16, 18, 20–22, 24, 33–35]. This study identified the risk factors for suboptimal response to aflibercept injection: longer DM duration, lower eGFR, higher serum creatinine and potassium levels, and the presence of ERM.

Previous epidemiological studies established that the prevalence of DME increases along with the duration of diabetes [13, 23]. Similar to our study results, DM duration has been reported as a negative predictor of anti-VEGF treatment response in DME [18, 36]. Eyes with a longer duration of DM are more likely to be chronically exposed to a DME-forming environment. It induces ongoing and irreversible macular tissue damage [37] and a transition from an acute inflammatory phase to a more treatment-resistant chronic inflammatory phase [38, 39]. Prompt anti-VEGF treatment in patients with DME results in more favorable outcomes than delayed treatment [40, 41]. Decreased eGFR levels are associated with hypercreatininemia [42] and hyperkalemia [43] as manifestations of renal dysfunction. Only a few studies have investigated the impact of renal dysfunction on the anti-VEGF treatment effects in DME. Consistent with our study, patients with lower eGFR levels tended to have poorer DME improvement after ranibizumab treatment [15]. Patients with higher serum creatinine levels were also prone to poorer visual improvement after bevacizumab treatment for DME [18]. In this study, the subgroup analysis revealed that mild to moderately decreased renal function, not severe renal failure, can even impact treatment response. We hypothesized that aflibercept treatment is less effective in DME patients with decreased renal dysfunction because both retinopathy and nephropathy are diabetes microvascular complications and share common systemic risk factors. Along with the epidemiological interrelationship between diabetic retinopathy and nephropathy, all renal anatomical endpoints were reported to be associated with increased severity of diabetic retinopathy [44], suggesting that renal dysfunction is an indicator of retinal vascular dysfunction. High levels of inflammation, vascular endothelial dysfunction, oxidative stress, and hypercoagulability have been proposed as common risk factors for diabetic retinopathy and nephropathy [44–46]. We postulated that osmotic imbalance induced by renal dysfunction is another contributing factor to persistent DME by interfering with fluid drainage from the retina. Fluid homeostasis in the retina is tightly regulated by water channels, such as aquaporins, in response to osmotic gradients by coupling with electrolyte channels located in the Muller glial cell and retinal pigment epithelium [47]. In addition, renal dysfunction may cause aflibercept to be less effective in DME as it increases serum VEGF levels. According to the previous study, the decrease in eGFR levels is associated with an elevated serum VEGF level in chronic kidney disease due to reduced elimination of VEGF and impaired oxygen delivery to tissues [34]. Reportedly, treatment of renal failure reduces macular edema in patients with both diabetic retinopathy and nephropathy [48].

Interestingly, our study showed that longitudinal changes in CRT and BCVA were similar but not completely consistent, suggesting that the anatomical resolution alone does not fully account for visual function. A post hoc analysis of Protocol T [49] revealed that retinal thickness and visual acuity have a small to moderate correlation during the follow-up. Furthermore, changes in retinal thickness only accounted for a small proportion (12-14%) of changes in visual acuity. A previous study indicated that macular ischemia in DME results in a discrepancy between anatomical and visual improvements after ranibizumab treatment [15]. These findings suggest that there would be more factors influencing visual acuity other than tissue edema in DME.

Inconsistent with our findings, some reported that there is no compelling evidence to suggest that systemic factors such as glycemic control, blood chemistry, or renal function are associated with treatment outcomes of ranibizumab [20, 22]. Meanwhile, others insisted that poor glycemic control (as evidenced by high HbA1c) [16, 20, 21, 24, 33, 35] is the major risk factor for poor anti-VEGF treatment outcomes. There is no clear answer to the inconsistency among the study results. However, we assume that it originated mainly from the differences in the study population, design, medication types, and observation period. It is important to note that all of our study patients had HbA1c levels < 8.0% due to the regulation of the Korean Health Insurance guidelines. Therefore, caution is needed in interpreting the results of our study. If we could include chronically uncontrolled DM patients in the analysis, the study results would differ from the current one. However, our study is important as it suggests that, even in patients with fairly good glucose control, controlling kidney dysfunction or serum electrolytes may potentially improve the response to aflibercept treatment.

In terms of ocular factors, similar to our study results, the presence of abnormalities at the vitreomacular interface, especially ERM, has been reported to be associated with poor DME treatment outcomes [14, 17]. A recent in vitro study revealed that this resistance of DME to anti-VEGF treatment is due to decreased antibody permeabilization through ERM [19].

The main limitation of this study was its retrospective design, which prevented the analysis of potential systemic risk factors, such as poor lipid profile, obesity, abnormal urinalysis, and smoking history. In addition, as this single-center study included only a small number of Korean patients, some inherent biases were inevitable. Unlike other studies that solely focus on treatment-naïve DME, our study included eyes with previous treatment histories. This heterogeneous study population would affect the study results, but it also reflects real-world clinical scenarios. A large-scale prospective multiracial study is required to support the results of this pilot study.

## 5. Conclusions

Five monthly loading doses of intravitreal aflibercept injection provided significant anatomical and visual improvements in patients with DME. Patients with long DM duration, renal dysfunction, or ERM were predisposed to a suboptimal treatment response. These systemic and ocular risk factors can predict the individual response of patients with DME to intensive aflibercept treatment.

## Figures and Tables

**Table 1:** Baseline characteristics of study patients.

Number of eyes	30 eyes of 23 patients
*Demographics factors*	Mean ± SD or *N* (%)
Age (years)	60.70 ± 9.28
Sex, male	19 (63.33)
Female	11 (36.66)
DM duration (years)	14.33 ± 8.73
HbA1C (%)	6.93 ± 1.52
Medication, insulin	4 (13.33)
OHA	26 (86.66)

*Ocular factors*	
DMR severity	
NPDR	18 (60.00)
PDR	12 (40.00)
Previous treatment history	
Vitrectomy+PRP	4 (13.33)
Bevacizumab+PRP	5 (16.66)
Bevacizumab only	5 (16.66)
PRP only	8 (26.66)
Treatment-naïve	8 (26.66)

SD: standard deviation; N: number; DM: diabetes mellitus; HbA1c: glycated hemoglobin; OHA: oral hypoglycemic agents; DMR: diabetic retinopathy; (N)PDR: (non)proliferative diabetic retinopathy; PRP: panretinal photocoagulation.

**Table 2:** Changes in central retinal thickness and best-corrected visual acuity in the whole cohort.

	Loading phase (monthly injection)					Maintenance phase (PRN regimen)							
	Baseline	1 mo	2 mo	3 mo	4 mo	5 mo	6 mo	7 mo	8 mo	9 mo	10 mo	11 mo	12 mo
CRT (*μ*m, mean ± SD)	486.87 ± 95.46	366.57 ± 75.54	338.30 ± 75.25	329.00 ± 57.49	331.27 ± 73.24	334.90 ± 69.47	390.74 ± 137.23	377.15 ± 110.89	390.45 ± 130.69	368.98 ± 58.81	391.00 ± 52.83	414.30 ± 97.49	382.00 ± 72.02
*p* value		0.021^∗^	0.028^∗^	0.014^∗^	0.014^∗^	0.011^∗^	0.042^∗^	0.022^∗^	0.010^∗^	0.010^∗^	0.036^∗^	0.168	0.012^∗^
BCVA (LogMAR, mean ± SD)	0.51 ± 0.30	0.41 ± 0.27	0.38 ± 0.28	0.36 ± 0.27	0.34 ± 0.24	0.35 ± 0.25	0.37 ± 0.26	0.36 ± 0.24	0.37 ± 0.26	0.39 ± 0.32	0.43 ± 0.18	0.38 ± 0.20	0.35 ± 0.23
*p* value		0.554	0.510	0.186	0.039^∗^	0.049^∗^	0.282	0.153	0.313	0.061	0.369	0.088	0.028^∗^

^∗^Statistically significant difference (*p* < 0.05), compared with preoperative values in repeated measures ANOVA. CRT: central retinal thickness; BCVA: best-corrected visual acuity; PRN: pro re nata; SD: standard deviation; mo: month.

**Table 3:** Comparison of baseline characteristics between good and suboptimal responders.

Mean ± SD or *N* (%)	Good responder	Suboptimal responder	*p* value
	(*n* = 11)	(*n* = 19)	
*Systemic factors*			
Age (years)	59.18 ± 10.34	61.58 ± 8.79	0.505
Sex, male	5 (45.45)	14 (73.68)	0.122
Female	6 (54.54)	5 (26.31)	
DM duration (years)	9.00 ± 7.04	17.42 ± 8.25	0.008^∗^
HbA1C (%)	7.01 ± 1.59	6.88 ± 1.53	0.834
Medication, insulin	1 (9.09)	3 (15.78)	0.603
OHA	10 (90.90)	16 (84.21)	
Hb (g/dL)	12.05 ± 3.56	10.82 ± 2.50	0.367
WBC (×10^3^/*μ*L)	7.44 ± 3.09	6.78 ± 2.95	0.616
Plt (×10^3^/*μ*L)	214.00 ± 106.58	214.42 ± 124.67	0.993
eGFR (mL/min/1.73 m^2^)	85.00 ± 25.88	53.80 ± 34.16	0.018^∗^
Cr (mg/dL)	1.03 ± 0.92	2.23 ± 1.98	0.049^∗^
Ca (mg/dL)	9.06 ± 0.88	9.43 ± 0.52	0.203
Na (mmol/L)	139.18 ± 2.68	134.19 ± 13.76	0.249
K (mmol/L)	4.20 ± 0.51	4.89 ± 0.60	0.004^∗^
Cl (mmol/L)	103.55 ± 3.14	107.38 ± 8.20	0.154
Alb (g/dL)	3.53 ± 0.89	3.87 ± 0.50	0.273
AST (IU/L)	25.27 ± 9.14	21.67 ± 10.17	0.361
ALT (IU/L)	24.27 ± 16.89	24.79 ± 17.31	0.941
HTN	3 (27.27)	10 (52.63)	0.177
HL	3 (27.27)	7 (36.84)	0.592

*Ocular factors*			
Baseline CRT (*μ*m)	470.73 ± 85.96	496.21 ± 101.61	0.490
Baseline BCVA (LogMAR)	0.43 ± 0.32	0.55 ± 0.28	0.293
DMR severity			0.643
NPDR	6 (54.54)	12 (41.37)	
PDR	5 (45.45)	7 (36.84)	
ERM	2 (18.18)	13 (68.42)	0.008^∗^
Previous treatment history			0.691
Vitrectomy+PRP	1 (9.09)	3 (15.78)	
Bevacizumab+PRP	2 (18.18)	3 (15.78)	
Bevacizumab only	1 (9.09)	4 (21.05)	
PRP only	3 (27.27)	5 (26.31)	
Treatment-naïve	4 (36.36)	4 (21.05)	

^∗^Statistically significant difference (*p* < 0.05) in Mann–Whitney *U*-test and Wilcoxon signed-rank test. SD: standard deviation; N: number; DM: diabetes mellitus; HbA1c: glycated hemoglobin; OHA: oral hypoglycemic agents; Hb: hemoglobin; WBC: white blood cell; Plt: platelet; eGFR: estimated glomerular filtration rates; Cr: creatinine; Ca: calcium; Na: sodium; K: potassium; Cl: chloride; Alb: albumin; AST: aspartate aminotransferase test; ALT: alanine aminotransferase test; HTN: hypertension; HL: hyperlipidemia; CRT: central retinal thickness; BCVA: best-corrected visual acuity; DMR: diabetic retinopathy; (N)PDR: (non)proliferative diabetic retinopathy; ERM: epiretinal membrane; PRP: panretinal photocoagulation.

## Data Availability

All data generated or analyzed during this study are available from the corresponding author on reasonable request.

## Supplementary Materials

Supplementary Materials Supplementary Table 1: cutoff values and odds ratio of the significant factors for suboptimal treatment response.
