# Supplementary material for: Factors Affecting Intensive Aflibercept Treatment Response in Diabetic Macular Edema: A Real-World Study
Source: J Diabetes Res. 2023 Jul 18;2023:1485059. doi: 10.1155/2023/1485059 (PMC10368507; doi:10.1155/2023/1485059)
Supplement: Supplementary Materials — Supplementary Table 1: cutoff values and odds ratio of the significant factors for suboptimal treatment response. [file 1485059.f1.pdf]

Supplementary Table 1. Cutoff values and odds ratio of the significant factors for suboptimal treatment response

	Cutoff value	OR	P-value
DM duration (years)	15	9.33	0.011*
eGFR (mL/min/1.73 m^2)	80	7.35	0.046*
Serum Cr (mg/dL)	0.95	7.33	0.026*
Serum K (mmol/L)	4.70	5.87	0.041*
ERM		9.75	0.014*

** Statistically significant ($p < 0.05$) in the logistic regression analysis*

OR, odds ratio; DM, diabetes mellitus; eGFR, estimated glomerular filtration rates; Cr, creatinine; K, potassium; ERM, epiretinal membrane
